# *In vitro* inhibitory effects of ganoderic acid A on human liver cytochrome P450 enzymes

**DOI:** 10.1080/13880209.2020.1747500

**Published:** 2020-04-14

**Authors:** Shangchen Xu, Fengqing Zhang, Dali Chen, Keren Su, Li Zhang, Rui Jiang

**Affiliations:** a Department of Neurosurgery, Shandong Provincial Hospital, Jinan, Shandong, China; b Department of Tumor Intervention, Municipal Official Hospital of WeiFang, Weifang, Shandong, China; c Department of Laboratory, Yidu Central Hospital of Weifang, Weifang, Shandong, China; d Department of Pharmacy, Shanxian Central Hospital (Affiliated Huxi Hospital of Jining Medical University), Heze, Shandong, China; e Department of Minimally Invasive Tumor, Shandong Provincial Hospital, Jinan, Shandong, China

**Keywords:** CYP3A4, CYP2D6, CYP2E1, drug-drug interaction

## Abstract

**Context:**

Ganoderic acid A (GAA) is usually used to prevent cancers or other diseases, which make it likely to be used with other drugs metabolized by cytochromes P450.

**Objective:**

This study investigates the effect of GAA on eight major cytochrome P450 isoforms in human liver microsomes.

**Material and method:**

The effects of GAA (100 μM) on eight human liver CYP isoforms (i.e., 1A2, 3A4, 2A6, 2E1, 2D6, 2C9, 2C19, and 2C8) were investigated *in vitro* using human liver microsomes (HLMs) with specific substrates for the CYPs, and the enzyme kinetic parameters were calculated.

**Results:**

The results showed that GAA inhibited the activity of CYP3A4, 2D6, and 2E1, but did not affect other isoforms. The inhibition of CYP3A4, 2D6, and 2E1 was concentration-dependent with *IC_50_* values of 15.05, 21.83, and 28.35 μM, respectively. Additionally, GAA was not only a non-competitive inhibitor of CYP3A4, but also a competitive inhibitor of CYP2D6 and 2E1, with *Ki* values of 7.16, 10.07, and 13.45 μM. Meanwhile, the inhibition of CYP3A4 was time-dependent, with the *K_I_/K_inact_* value of 7.91/0.048 μM/min.

**Discussion and conclusion:**

The *in vitro* study indicated that GAA has the potential to result in drug-drug interactions with other drugs metabolized by CYP3A4, 2D6, and 2E1. Further clinical studies are needed for the identification of this interaction.

## Introduction

Cytochrome P450 enzymes (CYPs) are one of the most important phase-I enzymes that participate in the metabolism of most clinical drugs in liver and intestine. The status of CYPs might influence the pharmacokinetics of co-administrated drugs and result in adverse effects, which would contribute to costly and late failures of drug development (Shi et al. 2016). The activity of CYPs is a notable factor during drug-drug interaction between different drugs in combination therapy. Previous studies demonstrated glycyrrhizic acid, verapamil, and grapefruit juice have effects on the pharmacokinetics of other drugs during the drug-drug interaction through inhibiting the activity of CYPs (Huang et al. 2018; Jia et al. 2018; Zhao et al. 2018). Moreover, numerous studies report CYPs are responsible for the bioactivation and inactivation of carcinogens and anticancer drugs (Rodriguez-Antona and Ingelman-Sundberg 2006; Mittal et al. 2015). For example, CYP2D6 is involved in the biotransformation of tamoxifen, which is widely used in the prevention and treatment of hormone-positive breast cancer (Dehal and Kupfer 1997). CYP2E1 acts an important role in the metabolism and activation of the carcinogens related to colorectal cancer (Jiang et al. 2013). Many drugs have been reported to have an inhibitory effect on the activity of CYPs, such as bergenin, isofraxidin and kaempferitrin (Dong et al. 2018; Song et al. 2019; Zhang et al. 2019).

*Ganoderma lucidum* (Leyss. Ex Fr.) Karst (Ganodermataceae), also called Ling Zhi in China, has been widely used in traditional Chinese medicine for more than 2000 years to promote health and longevity, as it has been reported to have anticancer and many other beneficial properties (Sliva 2004; Jiang et al. 2008; Zhu et al. 2018). Ling Zhi has also been used to prevent and treat various human diseases, and the main part of its extract ganoderic acid A (GAA), plays a vital role during the treatment. GAA has an inhibitory effect on the proliferation and invasion of hepatocellular carcinoma cells, and induce its apoptosis (Wang et al. 2017). GAA also has positive effect on the lung injury induced by lipopolysaccharide (Wan et al. 2019).

In addition to the medicinal application of *Ganoderma lucidum*, it has become a kind of common health care product. Therefore, GAA may interact with some drugs metabolized by CYPs in clinic therapy. However, the effect of GAA on the activity of CYPs has not been well documented, which is closely related to the bioavailability of drugs. This study investigates the effect of GAA on the eight major CYP isoforms in human liver microsomes. Through specific probe substrates: phenacetin (CYP1A2), testosterone (CYP3A4), coumarin (CYP2A6), chlorzoxazone (CYP2E1), dextromethorphan (CYP2D6), diclofenac (CYP2C9), *S*-mephenytoin (CYP2C19) and paclitaxel (CYP2C8), we clarified the effect of GAA on the activity of CYPs, and the inhibition model was determined by the enzyme kinetic studies.

## Materials and methods

### Chemicals

**Figure 1.**
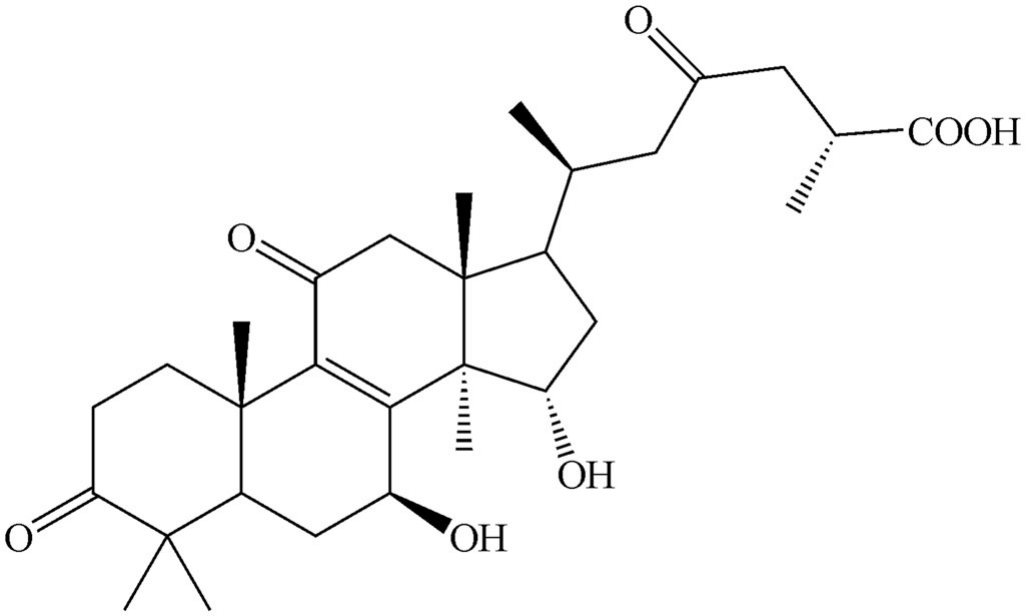
The chemical structure of GAA.

Ganoderic acid A (≥98%) and testosterone (≥98%) were obtained from the National Institute for the Control of Pharmaceutical and Biological Products (Beijing, China). The chemical structure of GAA is shown in Figure 1. d-Glucose-6-phosphate, glucose-6-phosphate dehydrogenase, corticosterone (≥98%), NADP^+^, phenacetin (≥98%), acetaminophen (≥98%), 4-hydroxymephenytoin (≥98%), 7-hydroxycoumarin (≥98%), 4′-hydroxydiclofenac (≥98%), sulfaphenazole (≥98%), quinidine (≥98%), tranylcypromine (≥98%), chlorzoxazone (≥98%), 6-hydroxychlorzoxazone (≥98%), paclitaxel (≥98%), 6β-hydroxytestosterone (≥98%), clomethiazole (≥98%), and furafylline (≥98%) were obtained from Sigma Chemical Co. Montelukast (≥98%) was obtained from Beijing Aleznova Pharmaceutical (Beijing, China). Coumarin (≥98%), diclofenac (≥98%), dextromethorphan (≥98%), and ketoconazole (≥98%) were purchased from ICN Biomedicals. Pooled HLMs were purchased from BD Biosciences Discovery Labware. All other reagents and solvents were of analytical reagent grade.

### Assay with human liver microsomes

As shown in Table 1, to investigate the effects of GAA on different CYP isoforms in HLM, the following probe reactions were used, according to previously described method (Zhang et al. 2007; Qi et al. 2013): phenacetin *O*-deethylation for CYP1A2, testosterone 6β-hydroxylation for CYP3A4, coumarin 7-hydroxylation for CYP2A6, chlorzoxazone 6-hydroxylation for CYP2E1, dextromethorphan *O*-demethylation for CYP2D6, diclofenac 4′-hydroxylation for CYP2C9, *S*-mephenytoin 4-hydroxylation for CYP2C19, and paclitaxel 6α-hydroxylation for CYP2C8. All incubations were performed in triplicate, and the mean values were utilized. The typical incubation systems contained 100 mM potassium phosphate buffer (pH 7.4), NADPH generating system (1 mM NADP^+^, 10 mM glucose-6-phosphate, 1 U/mL of glucose-6-phosphate dehydrogenase, and 4 mM MgCl_2_), the appropriate concentration of HLMs, a corresponding probe substrate and hispidulin (or positive inhibitor for different probe reactions) in a final volume of 200 μL.

**Table 1.** Isoforms tested, marker reactions, incubation conditions, and K_m_ used in the inhibition study.

CYPs	Marker reactions	Substrate concentration (μM)	Protein concentration (mg/mL)	Incubation time (min)	Estimated K_m_ (μM)
1A2	phenacetin *O*-deethylation	40	0.2	30	48
3A4	testosterone 6β-hydroxylation	50	0.5	10	53
2A6	coumarin 7-hydroxylation	1.0	0.1	10	1.5
2E1	chlorzoxazone 6-hydroxylation	120	0.4	30	126
2D6	dextromethorphan *O*-demethylation	25	0.25	20	4.8
2C9	diclofenac 4′-hydroxylation	10	0.3	10	13
2C19	*S*-mephenytoin 4-hydroxylation	100	0.2	40	105
2C8	paclitaxel 6α-hydroxylation	10	0.5	30	16

The concentration of GAA was 100 μM, and the positive inhibitor concentrations were as follows: 10 μM furafylline for CYP1A2, 1 μM ketoconazole for CYP3A4, 10 μM tranylcypromine for CYP2A6, 50 μM clomethiazole for CYP2E1, 10 μM quinidine for CYP2D6, 10 μM sulfaphenazole for CYP2C9, 50 μM tranylcypromine for CYP2C19, 5 μM montelukast for CYP2C8. Probe substrates, positive inhibitors (except for dextromethorphan and quinidine, which were dissolved in water) and GAA were dissolved in methanol, with a final concentration of 1% (v/v), and 1% neat methanol was added to the incubations without inhibitor. The final microsomal protein concentration and incubation times for the different probe reactions are shown in Table 1. There was a 3 min preincubation period (at 37 °C) before the reaction was initiated by adding an NADPH-generating system. The reaction was terminated by adding a 100 μL acetonitrile (10% trichloroacetic acid for CYP2A6) internal standard mix, and the solution was placed on ice. The mixture was centrifuged at 12,000 rpm for 10 min, and an aliquot (50 μL) of supernatant was transferred for HPLC analysis. The instrument used in this study was Agilent 1260 series instrument with DAD and FLD detector, and the quantitative assay for the corresponding metabolites was performed as previously reported (Lang et al. 2017; Zhang et al. 2017).

### Enzyme inhibition and kinetic studies of GAA

GAA (100 μM) was used to initially screen for its direct inhibitory effects towards different human CYP isoforms. For the CYP isoforms whose activities were strongly inhibited, secondary studies were performed to obtain the half inhibition concentration (*IC_50_*). *K_i_* values were obtained by incubating various concentrations of different probe substrates (20-100 μM testosterone, 10–50 μM dextromethorphan, 25–250 μM chlorzoxazone) in the presence of 0–50 μM GAA.

### Time-dependent inhibition study of GAA

To determine whether GAA could inhibit the activity of CYP3A4, 2D6, and 2E1 in a time-dependent manner, GAA (20 μM) was pre-incubated with HLMs (1 mg/mL) in the presence of an NADPH-generating system for 30 min at 37 °C. After incubation, an aliquot (20 μL) was transferred to another incubation tube (final volume 200 μL) containing an NADPH-generating system and probe substrates whose final concentrations were approximate to *K_m_*. Then, further incubations were performed to measure the residual activity. After being incubated for 10 and 30 min, the reactions were terminated by adding a 100 μL acetonitrile internal standard mix and then placed on ice; the corresponding metabolites were determined by HPLC.

To determine the *KI* and *K_inact_* values for the inactivation of CYP3A4, the incubations were conducted using higher probe substrate concentrations (approximately 4-fold *K_m_* values) and various concentrations of GAA (0–50 μM) after different preincubation times (0–30 min), with a two-step incubation scheme, as described above.

### Statistical analysis

The enzyme kinetic parameters for the probe reaction were estimated from the best fit line, using least-squares linear regression of the inverse substrate concentration versus the inverse velocity (Lineweaver-Burk plots), and the mean values were used to calculate *V_max_* and *K_m_*. Inhibition data from the experiments that were conducted using multiple compound concentrations were represented by Dixon plots, and inhibition constant (*K_i_*) values were calculated using non-linear regression according to the following equation:

$$v=\left(V_{\max}S\right)/\left(K_{m}\left(1+I/K_{i}\right)+S\right),$$

where I is the concentration of the compound, *K_i_* is the inhibition constant, S is the concentration of the substrate, and *K_m_* is the substrate concentration at half the maximum velocity (*V_max_*) of the reaction. The mechanism of the inhibition was assessed using the Lineweaver-Burk plots and the enzyme inhibition models. All data are represented as Mean ± SD. The data comparison was performed using Student’s *t*-test and performed using IBM SPSS statistics 20 (SPSS Inc.).

## Results

### Effect of GAA on the activity of CYPs

**Figure 2.**
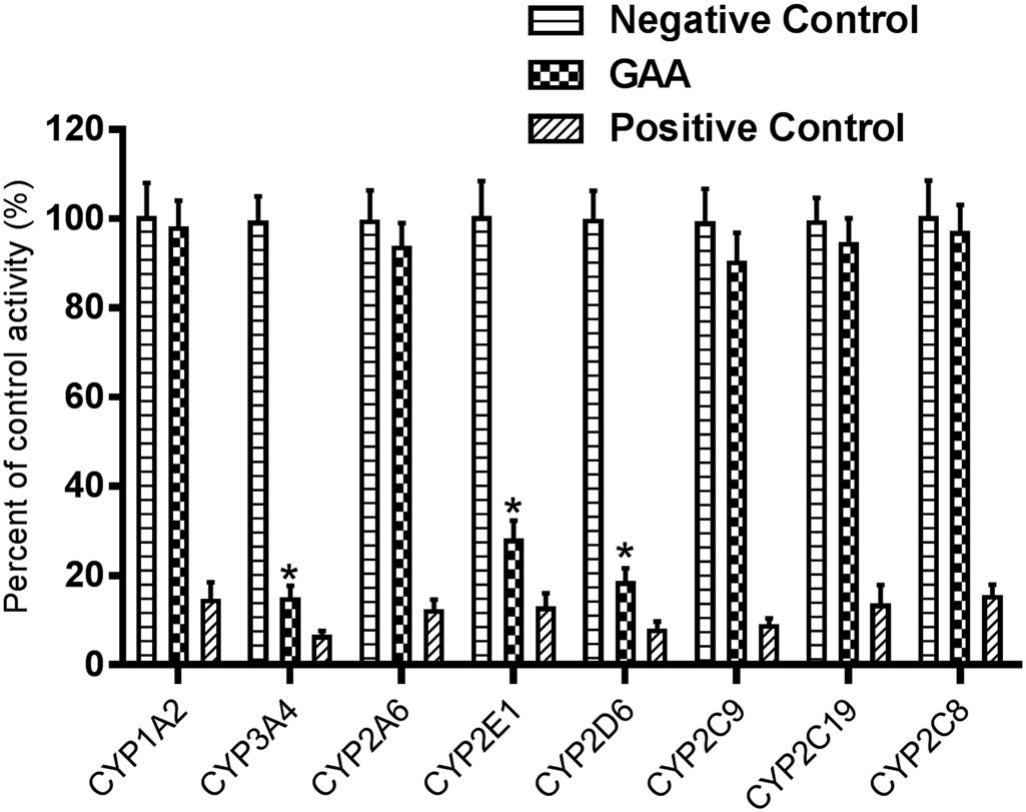
Inhibition of GAA on CYP enzymes in pooled HLMs. All data represent mean ± S.D. of the triplicate incubations. *p < 0.05, significantly different from the negative control. Negative control: incubation systems without GAA; GAA: incubation systems with GAA (100 μM); Positive control: incubation systems with their corresponding positive inhibitors.

Figure 2 shows the effect of GAA or specific inhibitors of CYPs on the activity of CYP1A2, CYP3A4, CYP2A6, CYP2E1, CYP2D6, CYP2C9, CYP2C19, and CYP2C8, with the employment of specific probe reaction assays. The results showed that GAA significantly inhibited the activity of CYP3A4, 2D6, and 2E1 to 14.6, 18.2, and 27.7% of their negative control, but did not exert effect on the activity of other CYPs. Compared with the positive control, the inhibitory effect of GAA was much weaker. Meanwhile, the inhibition of CYP3A4, 2D6, and 2E1 was concentration-dependent; whose *IC_50_* values were 15.05, 21.83, and 28.35 μM, respectively.

**Figure 3.**
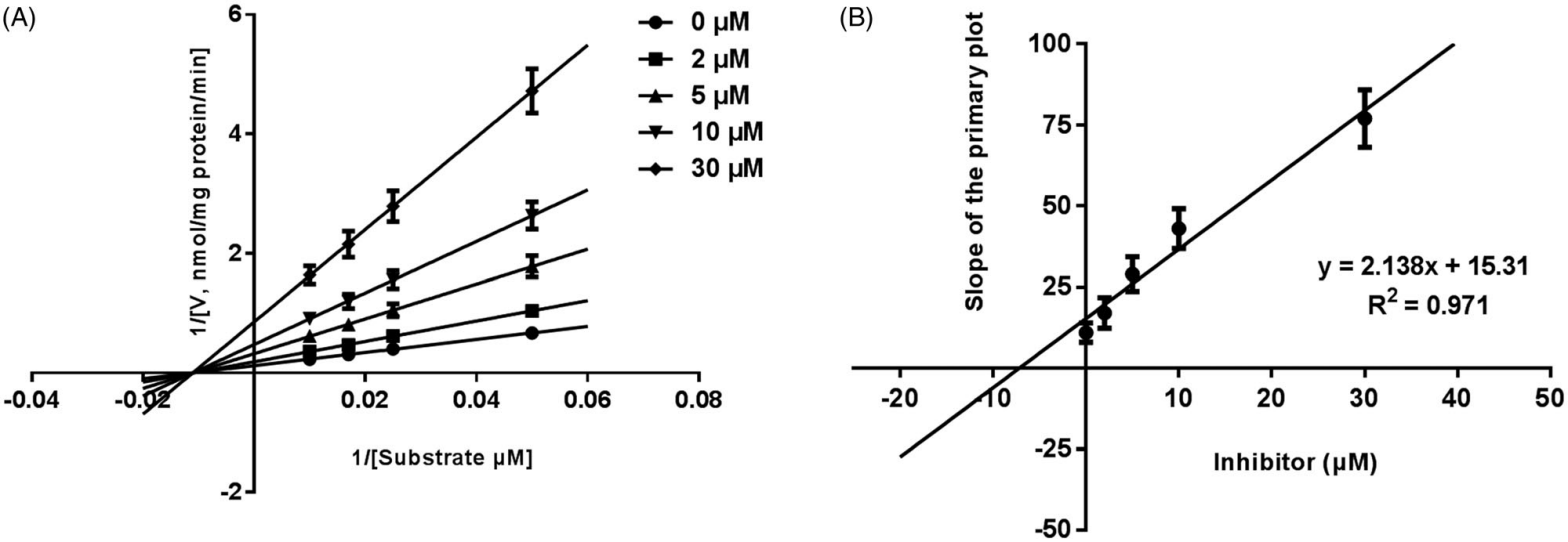
Lineweaver-Burk plots (A) and the secondary plot for *Ki* (B) of inhibition of GAA on CYP3A4 catalyzed reactions (testosterone 6β-hydroxylation) in pooled HLM. Data are obtained from a 30 min incubation with testosterone (20–100 μM) in the absence or presence of GAA (0–30 μM). All data represent the mean of the incubations (performed in triplicate).

**Figure 4.**
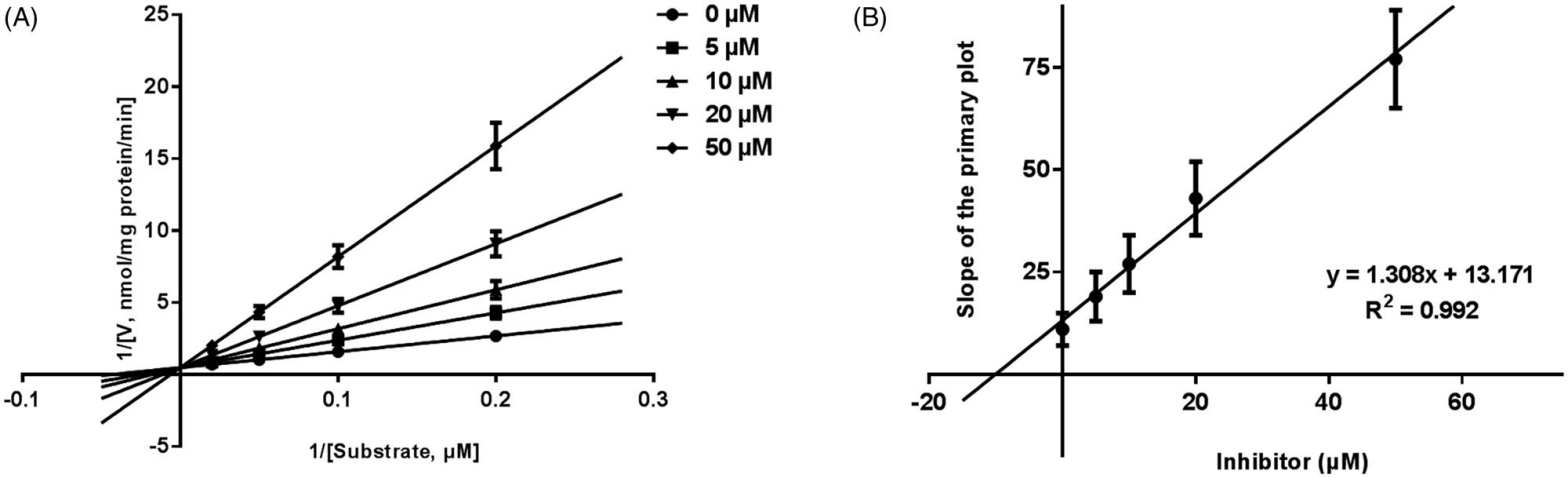
Lineweaver-Burk plots (A) and the secondary plot for *Ki* (B) of inhibition of GAA on CYP2D6 catalyzed reactions (diclofenac 4’-hydroxylation) in pooled HLM. Data are obtained from a 30 min incubation with dextromethorphan (10–50 μM) in the absence or presence of GAA (0–50 μM). All data represent the mean of the incubations (performed in triplicate).

**Figure 5.**
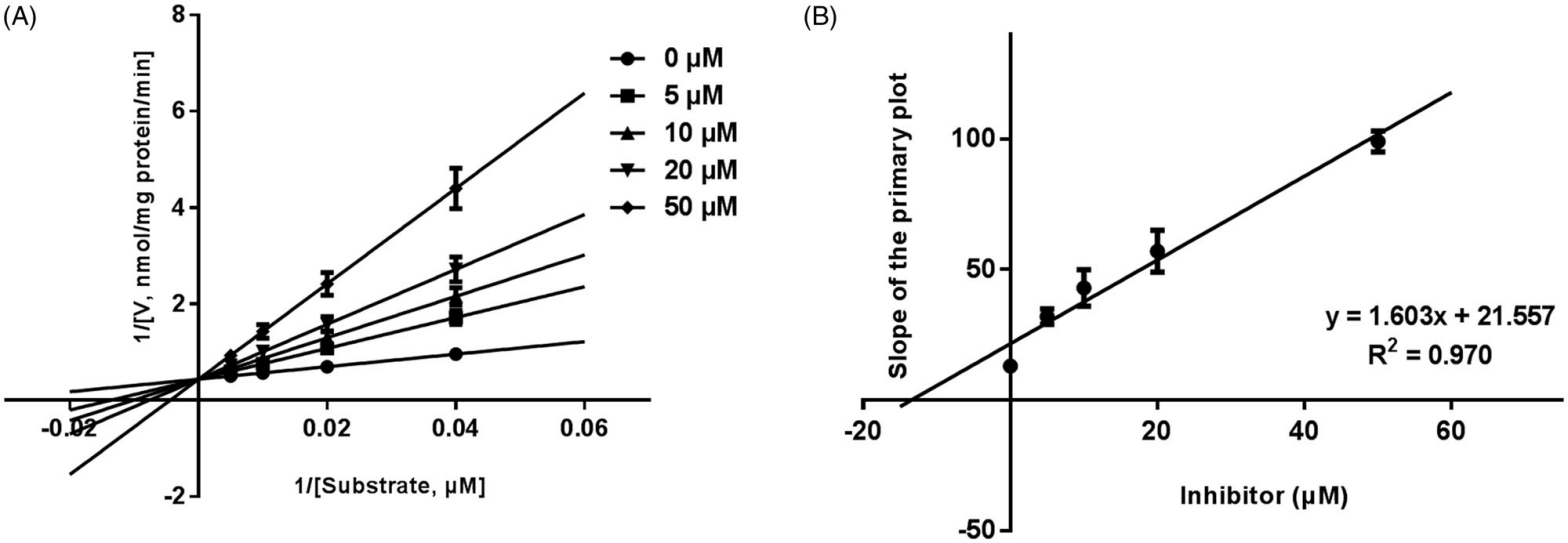
Lineweaver-Burk plots (A) and the secondary plot for *Ki* (B) of inhibition of GAA on CYP2E1 catalyzed reactions (chlorzoxazone 6-hydroxylation) in pooled HLM. Data are obtained from a 30 min incubation with chlorzoxazone (25–250 μM) in the absence or presence of GAA (0–50 μM). All data represent the mean of the incubations (performed in triplicate).

Next, the inhibition models of CYP3A4, 2D6, and 2E1 were investigated, and the Lineweaver-Burk plots of inhibitory kinetic data were shown in Figures 3–5. For CYP3A4 (Figure 3(A)), the inhibition of CYP3A4 was best fitted in a non-competitive manner, and after incubating 20, 40, 60, 100 μM testosterone in the presence of 0–50 μM GAA, the value of *K_i_* was obtained to be 7.16 μM (Figure 3(B)). The inhibition of CYP2D6 and CYP2E1 were performed competitively (Figures 4(A) and 5(A)), with the *K_i_* values of 10.07 and 13.45 μM, respectively (Figures 4(B) and 5(B)).

### Time-dependent inhibition

**Figure 6.**
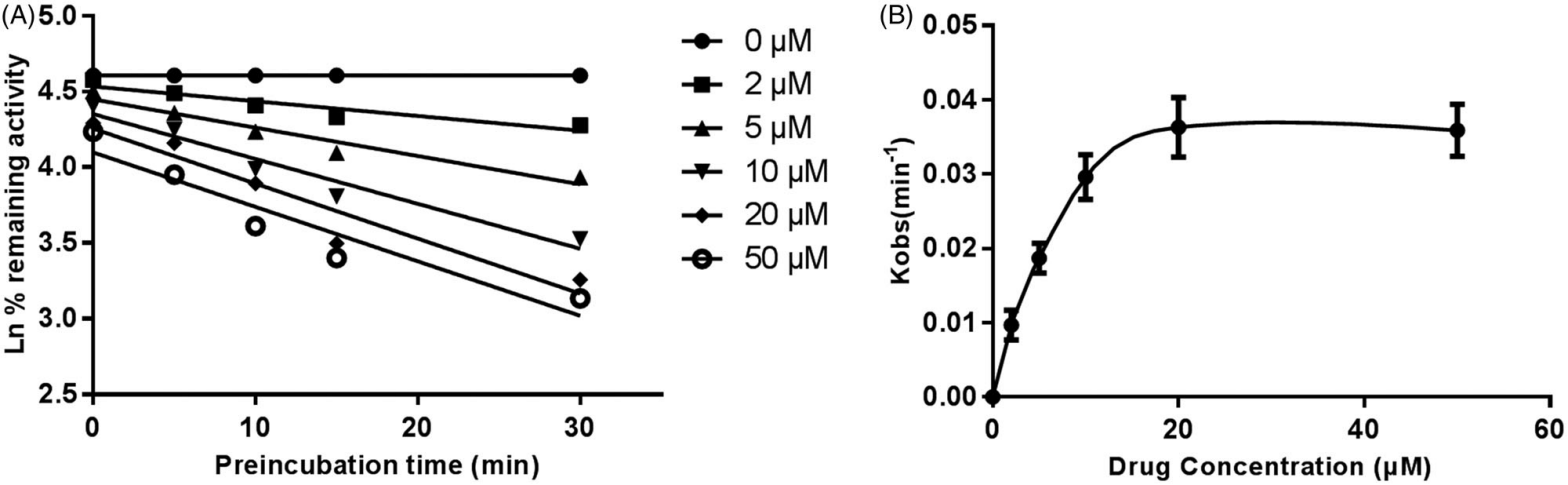
Time and concentration-inactivation of microsomal CYP3A4 activity by GAA in the presence of NADPH. The initial rate constant of inactivation of CYP3A4 by each concentration (*K_obs_*) was determined through linear regression analysis of the natural logarithm of the percentage of remaining activity versus pre-incubation time (A). The *K_I_* and *K*_*inact*_ values were determined through non-linear analysis of the *K_obs_* versus the GAA concentration (B).

The inhibitory effect of GAA on the activity of CYP3A4 performed time-dependent, as the inhibition become stronger with time. However, the inhibition of CYP2D6 and 2E1 was stable with incubation time. The time-dependent inhibition of CYP3A4 by GAA was further characterized through non-linear regression analysis, the result was shown in Figure 6. Moreover, the inactivation parameters of *KI* and *K_inact_* values were also calculated from the inactivation plot of Figure 6(B). The calculated *K_I_/K_inact_* value was 7.91/0.048 min/μM. From the value of *K_inact_*, it can be concluded that there are about 4.8% CYP3A4 was inactivated every minute, when GAA was incubated with HLM.

## Discussion

CYPs have been shown to be frequently involved in clinical drug-drug interaction, and during such interactions, the activity of CYPs is an important factor. While GAA is the main part of the extract of *Ganoderma lucidum*, it is inevitable that GAA may interact with other drugs metabolized by CYPs. Consequently, the present study investigated the activity of CYPs in the presence or absence of GAA, providing direct evidence for the effect of GAA on CYPs.

The results of the enzyme inhibition and kinetic studies showed the inhibitory effect of GAA on the activity of CYP3A4, 2D6, and 2E1. CYP3A is the most important drug metabolizing enzymes involved in drug clearance (Evans and Relling 1999). The CYP3A family of enzymes includes CYP3A4, 3A5, 3A7, and 3A4. Most of the CYP3A cleared drugs have been largely ascribed to the activity of CYP3A4, therefore, the effect on the activity of CYP3A4 will directly affect the clearance of most drugs metabolized by CYP3A. GAA inhibited the activity of CYP3A4 in a non-competitive manner, and the inhibition was increased with the incubation time. These results indicated that the dosage concentration and medication time of the drugs metabolized by CYP3A4 should be paid special attention, when combined with GAA. Moreover, among the CYP3A family, CYP3A5 can also contribute to the similar function to CYP3A4 (Lamba et al. 2002). Previous study has reported that some drugs or inhibitors which could exert inhibitory effect on the activity of CYP3A4, could also inhibit the activity of CYP3A5 (Jin et al. 2007; Tseng et al. 2014). Therefore, the drugs of which the metabolism is mediated by CYP3A5 should also be carefully used with GAA. Meanwhile, the effect of GAA on the activity of CYP3A5 needs further investigations to provide direct evidence.

Although the CYP2D6 isoform corresponds to only 2% of the CYPs in liver, it is also responsible for the metabolism of approximately 25% of drugs, which significantly increases the potential for drug-drug interaction (de Albuquerque et al. 2018). The inhibition of CYP2D6 could increase the oral bioavailability of various drugs and the risk of cancer (Mittal et al. 2015; Athukuri and Neerati 2016; Peterson et al. 2019). We have shown here that GAA inhibited CYP2D6-catalyzed dextromethorphan *O*-demethylation as a competitive inhibitor, though it is not a specific substrate of CYP2D6. It also indicated that the dose concentration of the CYP2D6-metabolized drugs could affect the inhibitory effect of GAA.

Additionally, GAA exerted similar effect on the activity of CYP2E1, which was also in a competitive manner. CYP2E1 accounts for 7% of total CYP450 enzymes in liver (Wang et al. 2009). Except mediating the metabolism of various drugs, CYP2E1 is also a powerful cellular pro-oxidant due to its strong oxidative potential (Cederbaum 2010). Previous studies have demonstrated that the increased level and activity of CYP2E1 were accompanied with the elevated ROS generation and intensification of peroxide oxidation (Linhart et al. 2014; Maksymchuk et al. 2017). The obtained results showed the inhibition of CYP2E1 by GAA, which may result in the decrease of cellular oxidation. And this result can explain the anti-oxidation property of GAA in some way. Certainly, the inhibition of CYP2E1 also suggested the potential drug-drug interaction, when the CYP2E1-metabolized drug co-administrated with GAA.

Actually, the *in vitro* inhibition cannot represent that the drug will cause clinically relevant interactions. There are many factors can affect the drug-drug interaction, including the contribution of the hepatic clearance during the metabolism of the drug, the fraction of the hepatic clearance which is subject to the inhibition of metabolism, and the ratio of the inhibition constant (*Ki*) over the *in vivo* concentration of the inhibitor (Ito et al. 1998; Ericsson et al. 2014). Therefore, further *in vivo* investigations are needed for the identification of the interaction between GAA and CYPs. In addition, according to previous studies, the maximum concentration in plasma of GAA was 2.24–10.99 ng/mL after oral administration of 3000 mg Lingzhi, which is much less than the IC_50_ values of CYP3A4, 2D6, and 2E1. (Teekachunhatean et al. 2012; Tseng et al. 2014). Therefore, the inhibitory effect of GAA may be very weak.

In conclusion, this paper investigated the effect of GAA on the activity of CYPs in HLM systemically. The results showed the inhibitory effect of GAA on the activity of CYP3A4, 2D6, and 2E1, while other isoforms were not affected. These results provided direct evidence for the activity of CYPs in the presence of GAA. And it is also recommended that GAA should be used carefully with other drugs metabolized by CYP3A4, 2D6, and 2E1.
